# OGG1 Inhibition Triggers Synthetic Lethality and Enhances The Effect of PARP Inhibitor Olaparib in BRCA1-Deficient TNBC Cells

**DOI:** 10.3389/fonc.2022.888810

**Published:** 2022-05-10

**Authors:** Juan Miguel Baquero, Erik Marchena-Perea, Rocío Mirabet, Raúl Torres-Ruiz, Carmen Blanco-Aparicio, Sandra Rodríguez-Perales, Thomas Helleday, Carlos Benítez-Buelga, Javier Benítez, Ana Osorio

**Affiliations:** ^1^ Human Genetics Group, Human Cancer Genetics Programme, Spanish National Cancer Research Centre (CNIO), Madrid, Spain; ^2^ Familial Cancer Clinical Unit, Human Cancer Genetics Programme, Spanish National Cancer Research Centre (CNIO), Madrid, Spain; ^3^ Molecular Cytogenetics Group, Human Cancer Genetics Programme, Spanish National Cancer Research Centre (CNIO), Madrid, Spain; ^4^ Division of Hematopoietic Innovative Therapies, Centro de Investigaciones Energéticas, Medioambientales y Tecnológicas (CIEMAT), Madrid, Spain; ^5^ Experimental Therapeutics Program, Spanish National Cancer Research Centre (CNIO), Madrid, Spain; ^6^ Science for Life Laboratory, Department of Oncology-Pathology, Karolinska Institutet, Stockholm, Sweden; ^7^ Sheffield Cancer Centre, Department of Oncology and Metabolism, University of Sheffield, Sheffield, United Kingdom; ^8^ Spanish Network on Rare Diseases (CIBERER), Madrid, Spain

**Keywords:** BRCA1, OGG1 inhibitor, PARP1 inhibitor, triple negative breast cancer, synthetic lethality

## Abstract

**Background:**

PARP1 plays a critical role in the base excision repair (BER) pathway, and PARP1 inhibition leads to specific cell death, through a synthetic lethal interaction, in the context of *BRCA1/2* deficiency. To date, up to five different PARP inhibitors (PARPi), have been approved, nevertheless, the acquisition of resistance to PARPi is common and there is increasing interest in enhancing responses and expand their use to other tumour types.

**Methods:**

We hypothesized that other BER members could be additional synthetic lethal partners with mutated BRCA genes. To test this, we decided to evaluate the glycosylase OGG1 as a potential candidate, by treating BRCA1 proficient and deficient breast cancer cells with PARPi olaparib and the OGG1 inhibitor TH5478.

**Results:**

Knocking out *BRCA1* in triple-negative breast cancer cell lines causes hypersensitivity to the OGG1 inhibitor TH5487. Besides, TH5487 enhances the sensitivity to the PARP inhibitor olaparib, especially in the context of *BRCA1* deficiency, reflecting an additive interaction.

**Discussion:**

These results provide the first evidence that OGG1 inhibition is a promising new synthetic lethality strategy in *BRCA1*-deficient cells, and could lead to a new framework for the treatment of hereditary breast and ovarian cancer.

## Introduction

Women who carry an inherited pathogenic *BRCA1* mutation present an increased risk of developing breast and ovarian cancer (1). BRCA1 is a multifunctional protein involved in repairing DNA double-strand breaks (DSBs) by the homologous recombination (HR) pathway (2). *BRCA1*-deficient tumour cells are sensitive to poly(ADP-ribose) polymerase (PARP1) inhibitors (PARPi) through a mechanism of synthetic lethality, an interaction in which inhibition of PARP1 results in loss of cell viability specifically in cells deficient in HR (3, 4). PARP1 has several roles in DNA repair, being essential for the detection and subsequent repair of single-strand breaks (SSBs), which are processed into DSBs during DNA replication (5). Consequently, due to the limited capacity for DSB repair in BRCA1-deficient cells, PARP inhibition triggers genomic instability and cell death (3, 6). Also, the inhibition of PARP, abolish its activity of auto-PARylation, a post-translational modification that allows PARP to be released from DNA damage sites causing a phenomenon called PARP trapping (7). The use of PARPi has become a promising therapeutic strategy for the treatment of BRCA deficient breast and ovarian cancer, and up to five different PARP inhibitors with different PARP-trapping efficiencies, have been approved for the treatment of specific breast or ovarian cancer subtypes, as single agents or in combination therapies with DNA damaging agents (7–10).

However, the fact that *BRCA*-mutated tumours frequently acquire resistance to PARPi through multiple mechanisms (11), together with the need to expand their use to other tumour types, has led to an active search for potential combinations with other drugs. It has been shown that the clinical use of PARPi in combination with conventional doses of chemotherapy regimens might be limited by the more-than-additive cytotoxicity (12), while combinations with antiangiogenics and immunotherapy might be safer and are being actively investigated (13). In light of this, the identification of additional synthetic lethal partners of *BRCA* genes, or the discovery of drug synergism with PARP inhibitors would represent promising therapeutic alternatives for cancer treatment (9). In this regard, the members of the base excision repair (BER) pathway have been considered as potential candidates (14), because PARP1 participates in the repair of the SSBs which are generated during this DNA repair pathway (15). In fact, it has been reported that inhibitors against the core BER enzymes APE1 and APE2 are synthetically lethal in BRCA1-deficient cells (16, 17). Nevertheless, bearing in mind that knockout mice for Ape1 are embryonic lethal, the treatment with APE1 inhibitors might cause unforeseen on-target toxicities in normal tissues (14). On the contrary, the deficiency of individual DNA glycosylases, which initiate BER, is relatively well-tolerated, and therefore these enzymes may be more promising candidates for drug development (14, 16, 18, 19). In particular, it has been described that the knockdown of OGG1 conferred sensitivity to PARP1 inhibition (20, 21).

A few years ago, TH5487, a small-molecule targeting OGG1, was developed and presented as a potential anti-inflammatory agent, as a consequence of the impairment of the interaction of OGG1 with the promoter regions of pro-inflammatory genes (22). Interestingly, the same authors have recently proposed OGG1 as an anti-cancer target, given that TH5487 has been shown to arrest cell proliferation and induce replication stress (23), and OGG1 inhibitors (OGG1i) are now considered as potential effective anti-cancer treatments (23, 24).

In addition, several genetic variants in glycosylase genes have been identified as cancer risk modifiers in *BRCA1/2* mutation carriers (25). In particular, the SNP rs2304277 which causes transcriptional down-regulation of the glycosylase OGG1 is associated with increased ovarian cancer risk for *BRCA1* mutation carriers (26).

Considering all these findings, we decided to evaluate the potential therapeutic impact of OGG1 inhibition on *BRCA1*-deficient cancer cells as well as its interaction with PARP inhibition. Here, we show that the knockout of *BRCA1* in triple-negative breast cancer (TNBC) cell lines increases their sensitivity to the OGG1 inhibitor TH5487, reflecting a possible synthetic lethal relationship between the two genes. Moreover, we have found that the synthetic lethality caused by the inhibition of PARP1 in *BRCA1*-deficient cells is enhanced when the PARPi Olaparib and TH5487 are combined.

## Methods

### Cell Culture and Treatments

The TNBC cell lines MDA-MB-231 (RRID: CVCL_0062) and Hs 578T (RRID: CVCL_0332) were purchased from ATCC (https://www.atcc.org/). Cells were cultured in DMEM (Lonza) growth medium supplemented with 10% of FBS (Biowest), 1% Penicillin-Streptomycin (Thermo Fisher Scientific) and 0.5% Amphotericin B (Thermo Fisher Scientific). All the cultures were carried out at 37°C in a 5% CO_2_ atmosphere. Cell line authentication was performed by STR profiling, and mycoplasma testing was performed regularly. To perform OGG1 inhibition, cells were released into a fresh medium containing the OGG1 inhibitor TH5487 (22). PARP inhibition was carried out incubating cells with olaparib (Axon Medchem). Stock solutions of TH5487 and olaparib were made in 100% Dimethyl sulfoxide (DMSO, Sigma-Aldrich) and working solutions were prepared in complete cell culture medium. Incubation periods and concentrations used of both inhibitors in the different studies are indicated in the corresponding figures. Complete cell culture medium with the same concentration of DMSO but without the tested inhibitor/s was used as control in treatments with TH5487 and/or olaparib.

Similar treatments were performed with the OGG1 inhibitor SUO268 and PARP inhibitor Niraparib (Selleck Chemical LLC) for validation of the results. All experiments were performed with mycoplasma-free cells. Cell lines have been authenticated using STR profiling.

### CRISPR/Cas9 Knockout of *BRCA1*

sgRNAs were designed using the Benchling CRISPR sgRNA Design tool (http://www.benchling.com). Specifics sgRNA were tested against the *BRCA1* gene (exon 11) and also a non-targeting control (NT) was used (sgBRCA1: GCTCATTACAGCATGAGAAC and sgNT: CCGCGCCGTTAGGGAACGAG). Those sequences were cloned into the lentiCRISPRv2 vector (plasmid #52961, Addgene) and verified by Sanger sequencing (primers listed in **Supplementary Table S1**). Viruses were produced by transient plasmid transfection into HEK293T cells by the calcium phosphate method, as previously described (27). Briefly, cells were seeded at 1.1 × 10^7^ cells/dish in 15-cm dishes the day before transfection. Cells were transfected using second-generation packaging plasmids (psPAX2 and pMD.2G, #12260 and #12259, respectively, Addgene) and the appropriate transfer plasmid (pLV CRISPR sgBRCA1 or sgNT). The medium was collected after 48 h, cleared by low-speed centrifugation, and filtered through 0.45 μm-pore-size PVDF filters (Millipore). Viral titers were calculated and values range around 107 to 108 TU/ml. In order to carry out transductions, cells were split and 24 h later were transduced using an MOI of 5 to ensure a high rate of transduced cells. Cells were incubated at 37°C for 12 hours. After that viral supernatant was replaced with a fresh cell medium.

**Figure 1:**
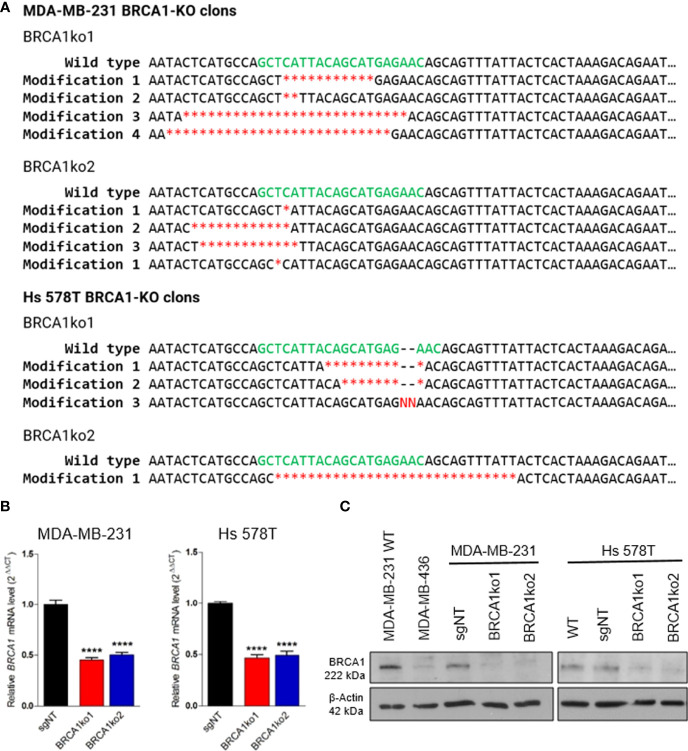
Validation of *BRCA1* knockout in BRCA-KO clones. **(A)** Deleted and inserted nucleotides in *BRCA1*-KO clones giving rise to premature termination codons in the *BRCA1* open reading frame. Sequences of guide RNA are indicated in green letters and red indicate nucleotide deletions (*) or insertions (N). **(B)** *BRCA1* mRNA relative level in parental non-targeting control TNBC cells (sgNT), and two *BRCA1*-KO clones (BRCA1ko1 and BRCA1ko2). **(C)** Western blotting of BRCA1. β-actin was used as a loading control. Unpaired t‐tests were used in **(B)**. Bars show the mean and the SEM of three independent experiments. *p-value<0.05; **p-value<0.01; ***p-value<0.001 and ****p-value<0.0001.

*BRCA1* knockout cells were generated using the MDA-MB-231 and Hs 578T TNBC cell lines. Several single colony clones were established, some of which displayed reduced *BRCA1* mRNA expression. Then, these clones were selected for Western blotting validation. *BRCA1* knockout clones were validated by Sanger sequencing of the targeted region, followed by analysis using Tracking of Indels by Decomposition (TIDE) (https://tide.nki.nl), confirming *BRCA1* gene disruption (**Figure 1A**).

### DNA, RNA Extraction, cDNA, qPCR

DNA and RNA were extracted from cultured cells using the DNeasy^®^ Blood & Tissue Kit (Qiagen) or TRIzol^®^ Reagent (Thermo Fisher Scientific), respectively. In both cases, according to the manufacturer´s instructions. Subsequently, extracted DNA was quantified by the PicoGreen^®^ fluorometric assay (Thermo Fisher Scientific). RNA quantity and quality were assessed by NanoDrop^®^ (Thermo Fisher Scientific).

The High Capacity cDNA Reverse Transcription Kit (Applied Biosystems) was utilized for cDNA synthesis following the manufacturer’s instructions using 1000 ng of total RNA. Two μL of cDNA at a final concentration of 10 ng/μL was mixed with 1 x GoTaq^®^ qPCR MasterMix (Promega) and 1 μM cDNA primers of each pair of primers (F/R) in a final volume reaction of 10 μL. Primers used are listed in **Supplementary Table S1**. The amplification conditions consisted of an initial step at 95°C for 10 min, followed by 40 cycles of 10 s at 95°C and 1 min at 65°C. Each qPCR was performed in triplicate including no-template controls in an ABI QuantStudio S6 Flex System (Applied Biosystems). Relative genes mRNA expression was calculated using the 2ΔΔCt method for qPCR analysis after normalization with the housekeeping gene *GAPDH* using the QuantStudioTM Real-Time PCR Software (Applied Biosystems).

### Protein Extraction and Western Blotting

Protein expression was determined by Western blotting. Briefly, cell pellets of cultured cells were lysed in RIPA buffer (Sigma) in the presence of Complete Protease Inhibitor Cocktail (Roche). Total protein concentration was determined using the Pierce BCA Protein Assay Kit (Thermo Fisher Scientific) following the manufacturer’s instructions. Sixty micrograms of protein were electrophoresed on 12% SDS/PAGE and transferred to Immobilon-FL membranes (Millipore). Membranes were blocked in TBS-T (50 mM Tris-HCl, 150 mM NaCl, pH 7.5 plus 0.1% Tween-20) and 5% non-fat milk for 1 h at room temperature. Blots were probed over-night at 4 °C with the following primary antibodies: rabbit anti-BRCA1 (sc-6954; Santa Cruz) at 1/200 dilution and mouse anti-β-Actin (A5441; Sigma) at 1/10,000 dilution in blocking solution. Anti-mouse and anti-rabbit IgG-HRP (Dako) were used as the secondary antibodies at 1/10,000 dilution in blocking solution 1 h at room temperature. Immunoblots were developed using Immobilon Classico Western HRP substrate (Millipore). Each western blot was performed at least in duplicate. Images were analyzed using ImageJ software (NIH Image) and BRCA1 protein levels were normalized by β-Actin.

### Colony Formation Assay

**Figure 2:**
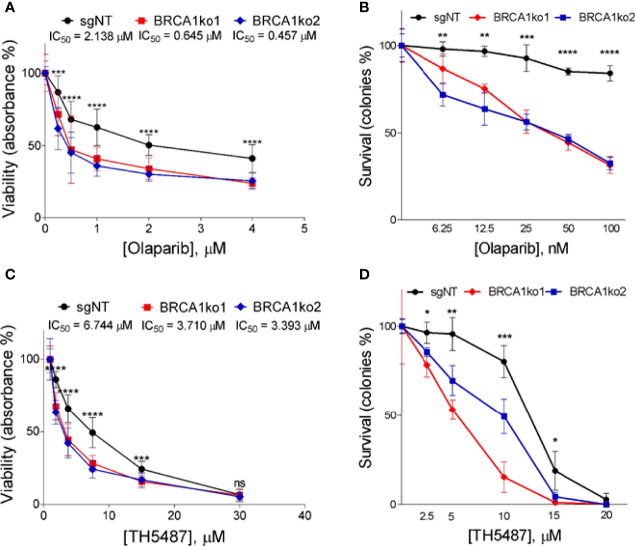
*BRCA1* knockout sensitizes MDA-MB-231 TNBC cell line to PARP1 and OGG1 inhibition. **(A)** MTT assay displaying logarithm-transformed values and the viability curves of BRCA1-proficient (sgNT) and deficient (BRCA1ko1 and BRCA1ko2) MDA-MB-231 cells after treatment with olaparib for 72 hours. **(B)** Clonogenic survival of BRCA1-proficient (sgNT) and deficient (BRCA1ko1 and BRCA1ko2) MDA-MB-231 cells exposed to olaparib. **(C)** MTT assay displaying drug doses used and the viability curves of BRCA1-proficient (sgNT) and deficient (BRCA1ko1 and BRCA1ko2) MDA-MB-231 cells after treatment with TH5487 for 72 hours. **(D)** Clonogenic survival of BRCA1-proficient (sgNT) and deficient (BRCA1ko1 and BRCA1ko2) MDA-MB-231 cells exposed to TH5487. In **(A, C)** for each concentration were included six replicates in at least two independent plates. IC_50_ calculated based on the resulting dose-response curves are shown. In **(B, D)** cells were incubated for 14 days in the presence of DMSO (control) or the indicated concentrations of olaparib or TH5487 in three independent experiments. In **(A–C)**, and **(D)** statistical significance at each olaparib or TH5487 concentration was determined by a one-way ANOVA test. The values are normalized to untreated cells and error bars represent one standard deviation around the mean. *p-value<0.05; **p-value<0.01; ***p-value<0.001 and ****p-value<0.0001. ns, not significant.

Cells (sgNT or KO clones) were seeded at a density of 350 cells/well in 6-well plates. Twenty-four hours after seeding, the medium was replaced and cells were treated with PARP inhibitor olaparib and/or OGG1 inhibitor TH5487 and were incubated until colony size surpassed a minimum of 50 cells (≈12-14 days). The different concentrations used for both inhibitors are indicated in the corresponding figures (**Figures 2B, D** and **Supplementary Figures 1B, D**). Complete growth medium with the same concentration of DMSO as the one used in the highest concentration for each inhibitor but without the tested inhibitor/s was used as control. Finally, cells were washed twice with PBS, fixed with ice-cold methanol (Sigma) for 10 min, and stained with 1% crystal violet solution (Sigma) for 20 min, followed by extensive washes in tap water and air drying. The plates were scanned and the number of colonies per well was measured with ImageJ software (NIH Image).

### MTT Colorimetric Assay

The effect of PARP and OGG1 inhibition on cell viability was assessed in the TNBC cell lines using the MTT colorimetric assay. Cells were seeded in 96-well plates at a density of 2,500 cells per well and, after 8 h, treated with olaparib, TH5487, niraparib, SUO268 or a combination of drugs at different concentrations for 72 h. The different concentrations used for both inhibitors are indicated in the corresponding figures (**Figures 2A, C** and **Supplementary Figures 1A, C**, **2**). Six replicates for each concentration were used, with a 1% DMSO final concentration, in at least two independent plates. MTT (Sigma-Aldrich) dissolved in PBS was added to a final concentration of 1 g/l and incubated 4 h at 37 °C. Afterwards, the medium was removed and 50 μL of 100% DMSO were transferred into each well to dissolve the formazan crystals. Compounds were added to the plates using the Biomeck NPX Laboratory Automation Workstation (Beckman Coulter). Absorbance at 544 nm was read on a spectrophotometer (VICTOR Multilabel Plate Reader; PerkinElmer). The data were normalized to a mean absorbance detected in wells containing media without cells, and the results were expressed as a percentage (%) of the control (DMSO-treated cells). Curves were fitted using GraphPad Prism 8 (GraphPad Software Inc) and half-maximal inhibitory concentration (IC_50_) values were determined.

### Drug Combination Effect Analysis

Combination effect between drugs was evaluated by calculating the combination index (CI) based on the Bliss Independence model (28) whereby the CI was calculated with the following equation: CI=Ea+Eb-Ea*Eb/Eab, where Ea indicates the viability effect of drug A, Eb indicates the viability effect of drug B and Eab indicates the viability effect of the drug combination. CI < 1 indicates synergism, CI = 1 indicates additivity and CI > 1 indicates antagonism.

### Evaluation of DNA Damage by Confocal Microscopy

γH2AX mean signal intensity in the TNBC cell lines was measured as a marker of DNA damage. Cells were seeded in uCLEAR bottom 96‐well plates (Greiner Bio‐One) at a density of 2,500 cells per well and, after 8 h, treated with olaparib, TH5487, or a combination of drugs at different concentrations for 72 h, followed by the following immunofluorescence protocol. Cells were fixed with 4% paraformaldehyde (PFA, Agar Scientific) for 10 min. After washing with PBS (Sigma), cell permeabilization was performed with 0.5% Triton X-100 in PBS for 15 min. Blocking with 3% BSA (Sigma) in PBS for 1 h was followed by staining with primary (overnight) and secondary antibodies (1 h) and 0.5 μg/ml 4′,6-Diamidino-2-Phenylindole (DAPI; Sigma) to visualize nuclei. After each staining, a washing step was performed three times (10 min in PBS each). All steps were performed at room temperature. Antibodies used were primary rabbit anti-phospho-histone H2AX (#9718, Cell Signalling) and secondary anti-rabbit Alexa 555 (TermoFisher Scientific). Images were automatically acquired from each well using an Opera High‐Content Screening System (Perkin Elmer). Images were segmented based on the DAPI staining to generate masks matching cell nuclei, from which mean signal intensities were calculated. We considered γH2AX positive cells those with a pan-nuclear γH2AX signal intensity higher than an arbitrarily chosen threshold of 500 arbitrary units.

### Statistical Analysis

The Kolmogorov-Smirnov test was used to evaluate if the data sets were normally distributed. For comparative analyses between two groups of data, statistically significant differences were assessed by Student´s unpaired t-test for normally distributed variables and the Mann-Whitney U test for non-normal data distribution. For comparative analyses between three or more groups, statistically differences were analyzed with the one-way analysis of variance (ANOVA) test. Statistical calculations and graphs were done using GraphPad Prism 8 (GraphPad Software Inc). In all analyses, a 2-tailed p-value <0.05 was considered statistically significant: *P<0.05; **P<0.01; ***P<0.001 and ****P<0.0001.

## Results

### *BRCA1* Knockout Sensitizes TNBC Cells to PARP1 and OGG1 Inhibition

TNBC cell lines MDA-MB-231 and Hs 578T were selected for this study, first because this phenotype is frequently associated to the lack of *BRCA1*, and we thought that they could best represent a *BRCA1*-deficiency context, and secondly because TNBC is the most aggressive subtype and presents limited choices of therapy (29, 30). We used CRISPR/Cas9 to silence the *BRCA1* gene in these cell lines and *BRCA1* knockout (KO) single colony clones were generated. Gene disruption in the selected clones was confirmed by DNA sequencing (**Figure 1A**). Additionally, the quantitative RT-PCR analysis showed a significantly lower *BRCA1* mRNA expression in *BRCA1*-KO clones compared to non-targeting control cells (**Figure 1B**), and Western blotting confirmed BRCA1 protein loss (**Figure 1C**).

Firstly, in order to validate the synthetic lethal interaction between *BRCA1* and *PARP1* (3) we treated BRCA1-proficient (sgNT) and *BRCA1*-KO clones with the PARP inhibitor olaparib and analysed differences in cell proliferation and the clonogenic potential. As expected, *BRCA1* knockout markedly increased sensitivity to olaparib. In both cell lines, *BRCA1*-KO clones showed a decrease in cell proliferation (**Figure 2A** and **Supplementary Figure 1A**) and a significantly relative lower number of colonies compared to BRCA1-proficient cells (**Figure 2B** and **Supplementary Figure 1B**) in the full range of olaparib concentrations tested. These results confirm that PARP inhibition causes a selective loss of viability in *BRCA1*-deficient TNBC cells.

As is the case of PARPi, we decided to evaluate whether *BRCA1* knockout sensitizes TNBC cells to OGG1 inhibition. To this end, we incubated the TNBC BRCA1-proficient and their derived *BRCA1*-KO clones with a dilution series of the OGG1 inhibitor TH5487 (22) up to 72 h to analyse differences regarding cell proliferation. *BRCA1*-KO clones displayed slower proliferation for up to 72 h and present substantially lower IC_50_ values for TH5487 than control cells (**Figure 2C** and **Supplementary Figure 1C**). In parallel, we also found that TH5487 decreased significantly clonogenic potential in *BRCA1* knockout clones compared to BRCA1-proficient cells (**Figure 2D** and **Supplementary Figure 1D**). Overall, these data show that *BRCA1* knockout increases sensitivity to OGG1 inhibition, supporting that a synthetic lethal interaction might exist between *BRCA1* and *OGG1*. We further validated these findings by treating one of the KO clones of Hs 578T (BRCA1ko2) with another OGG1 inhibitor, SUO268, for which we obtained a IC50 of 6.64µM in the KO compared to 11.29µM in the BRCA1-proficient cell in the cell viability assays (**Supplementary Figure S2**). This confirms that the synthetic lethal interaction between *BRCA1* and *OGG1* inhibition is not an off-target effect.

### PARP1 and OGG1 Inhibition Combined Treatment Has Greater Effect Than Single Drugs in BRCA1-Deficient Cells

**Figure 3:**
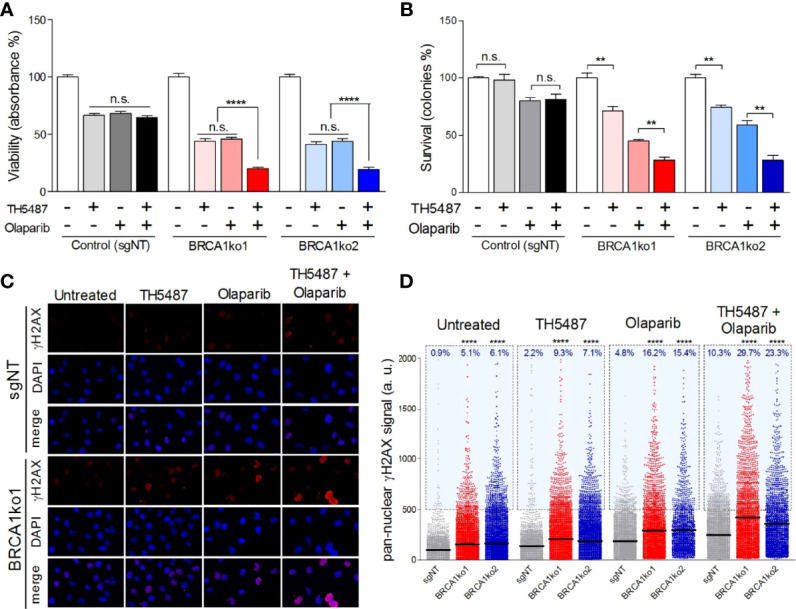
OGG1 inhibition enhances the sensitivity to olaparib in BRCA1-deficient MDA-MB-231 cells. **(A)** Cell viability assessment using MTT displaying proliferation changes of BRCA1-proficient (sgNT) and deficient (BRCA1ko1 and BRCA1ko2) MDA-MB-231 cells after single-drug (TH5487 3.75 µM or olaparib 0.5 µM) or combined treatments for 72 hours. **(B)** Clonogenic survival of BRCA1-proficient (sgNT) and deficient (BRCA1ko1 and BRCA1ko2) MDA-MB-231 cells exposed to TH5487 (3 µM), olaparib (30 nM), or a combination of both inhibitors. **(C)** Immunofluorescent staining of γH2AX. A representative confocal image example of one of three independent experiments is shown for control (sgNT) and BRCA1ko1 cells for each treatment condition. Cells were stained for γH2AX (red) and DAPI (blue) was used to stain cell nucleus. **(D)** Pan-nuclear γH2AX signal intensity of BRCA1-proficient (sgNT) and deficient (BRCA1ko1 and BRCA1ko2) MDA-MB-231 cells exposed to TH5487 (3.75 µM), olaparib (0.5 µM), or a combination of both. In **(A, B)** values are normalized to untreated cells (DMSO) and bars represent the mean and the SEM of at least three independent experiments. In **(D)** each dot represents the signal from one cell, horizontal lines indicate mean values, and the blue area delineates cells above an arbitrarily chosen threshold (500 arbitrary units of pan-nuclear γH2AX signal intensity). Each condition includes at least 2000 cells from 3 independent experiments. Unpaired t‐tests were used in **(A, B)**. In **(D)** statistical significance was determined by Mann-Whitney U tests. **p-value<0.01 and ****p-value<0.0001. ns, not significant.

Considering the two synthetic lethal interactions found between *BRCA1* and the BER members *OGG1* and *PARP1*, we decided to analyse the possible interaction between the inhibitors of these two BER enzymes in the context of BRCA1 deficiency. To test this, we selected sublethal concentrations of olaparib (0.5 µM and 30 nM in MTT and colony formation assays, respectively) and TH5487 (3.75 µM and 3 µM in MTT and colony formation assays, respectively), which caused a significantly higher impact on the *BRCA1*-KO clones than BRCA1-proficient TNBC cells, and evaluated their effect on cell proliferation and clonogenic potential. According to the previous experiments, single treatments with both inhibitors at the chosen concentrations induced a significantly higher decrease in proliferation and clonogenic potential in the *BRCA1-*KO clones (**Figures 3A, B**, and **Supplementary Figures S1E, F**). Interestingly, the combination of both inhibitors significantly decreased cell proliferation by a greater extent than each of the treatments alone in *BRCA1*-depleted clones, while in BRCA1-proficient cells, TH5487 did not enhance the effect of the olaparib treatment alone (**Figure 3A** and **Supplementary Figure S1E**). Furthermore, the combined treatment with these two inhibitors notably diminishes the number of colonies compared to single-drug treatments in *BRCA1*-KO clones but not in the control cells (**Figure 3B** and **Supplementary Figure S1F**). A formal analysis of the interaction was performed by calculating the combination index (CI) based on Bliss model showing an additive effect between the drugs in both cell lines for the viability assays, which reached values of synergy for the clonogenic survival analysis (**Supplementary Table 2**) in the BRCA1-deficient context. These results support that the OGG1 inhibitor TH5487 increases the sensitivity to PARP1 inhibition especially in BRCA1-depleted TNBC cells. When combining the alternative PARP and OGG1 inhibitors Niraparib and SUO268, a synergic effect was seen in both the parental and *BRCA1*-KO cell lines. This probably reflects the off target effect of kinase inhibition recently described to be caused by niraparib (31), while being olaparib much more specific to inhibit PARP, the effect would only be seen in a BRCA1-deficient context (**Supplementary Table 2**).

Finally, we aimed to gain a better understanding of the molecular mechanisms behind the putative synthetic lethal interaction found between OGG1 and BRCA1. We hypothesized that the inhibition of OGG1 could promote the accumulation of oxidative DNA lesions that may progress to SSB and, similarly to PARP inhibition (3), triggering genomic instability with lethal consequences in BRCA1-deficient cells. To analyse this premise, we assessed by high-throughput microscopy the pan-nuclear phosphorylated H2AX (γH2AX) staining as a commonly used indicator of DSBs (32) and replication stress (33), in TNBC BRCA1-proficient cells and their derived *BRCA1*-KO clones. Under basal conditions, *BRCA1*-KO clones showed a significantly higher level of mean γH2AX signal intensity as well as a higher percentage of γH2AX positive cancer cells (**Figures 3C, D**, and **Supplementary Figure S1G**). Next, we incubated the cells with the already determined sublethal concentrations of olaparib and TH5487 for 72 hours. Interestingly, olaparib and TH5487 combined treatment increases the mean γH2AX signal intensity in both BRCA1-proficient and deficient cells, but the level of DNA damage was significantly more elevated in *BRCA1*-KO clones (**Figures 3C, D**, and **Supplementary Figure S1G**). This suggests that OGG1 inhibition, just like PARP1 inhibition, can lead to DSBs generation, especially in the context of BRCA1 deficiency. Finally, we assessed the molecular consequences caused by the synergistic interaction between olaparib and TH5487. The combined treatment generates higher γH2AX signal intensity values than single-drug treatments both in BRCA1-proficient and deficient cells, indicating that OGG1 inhibition intensifies the accumulation of DNA damage caused by the treatment with olaparib (**Figures 3C, D**, and **Supplementary Figure S1G**). Furthermore, the effect on DNA damage attributed to the combined treatment was significantly more pronounced in BRCA1-deficient clones. These results indicate that the impact on cell viability of PARP inhibition enhanced by TH5487 in BRCA1-deficient cells can be, at least, partially due to the accumulation of DNA damage resulting in selective cell death.

## Discussion

The dependence on compensatory DNA repair pathways in cancer cells can be exploited as a therapeutic strategy in cancer therapy (34). Considering that BRCA1-deficient cancer cells are hypersensitive to the inhibition of the BER component PARP1, we decided to analyse the potential therapeutic use of the OGG1 inhibitor TH5487 (22) specifically in the context of BRCA1 deficiency. Here, we have found that *BRCA1* knockout in TNBC cell lines increase their sensitivity to OGG1 inhibition with TH5478, providing the first evidence that OGG1 inhibition is a promising new synthetic lethality strategy in BRCA1-deficient cells.

Several reports have shown that the most direct consequence of OGG1 inhibition or depletion is the accumulation of oxidative DNA damage (35, 36). This accumulation of 8-oxoG is highly mutagenic (37) and triggers distinct pathways of cell death (38). Thus, considering that both OGG1 and BRCA1 contribute to reducing intracellular oxidative stress (OS) (21), we hypothesized that OGG1 inhibition might generate more elevated reactive oxygen species (ROS) levels than BRCA1-deficient cells can handle. Besides, *OGG1* knockout and TH5487 treatment induce alterations in the expression of multiple genes, evidencing that OGG1 acts as a modulator of gene expression (22, 23). In particular, TH5487 decreases proinflammatory gene expression (22), which could represent an alternative mechanism of action of this molecule that contributes to explaining the observed results. Therefore, apart from leading to excessive ROS accumulation, OGG1 inhibition could decrease the expression of certain essential genes for BRCA1-deficient cells survival. Interestingly, OGG1 binds to PARP1, stimulating its poly ADP-ribosylation activity and OGG1 knockout cells show decreased poly ADP-ribose levels compared with wild-type cells (39). Hence, the severe effects of OGG1 inhibition in the context of BRCA1 deficiency may result from indirect PARP1 inhibition.

On the other hand, taking into consideration both synthetic lethal interactions of *BRCA1* with *PARP1* and *OGG1*, we combined PARP1 and OGG1 inhibitors to study their possible joint effects on BRCA1-deficient cells. We found that, especially for the *BRCA1*-knockout clones, the combined treatment significantly decreased cell viability and the clonogenic potential compared to single-drug treatments. A recent study has suggested that OGG1 inhibition would mitigate the impact of PARPi by preventing the formation of SSBs which are processed into DSBs during DNA replication, being particularly cytotoxic for BRCA1-deficient cells (40). In contrast to this, our results show that TH5487 -alone or combined with olaparib- increases γH2AX signal, reflecting the generation of DSB as a consequence of OGG1 inhibition, and thus supporting the impairment of DNA damage repair as the principal mechanism underlying the greater effect seen when combining TH5487 and olaparib. There are important differences between both study designs that can explain the discordant results, being the main one that in (40), the treatments with TH5478 and olaparib are induced sequentially while in our case they are simultaneous. Moreover, for the doss of TH5478 used for the clonogenic assays in (40) (0.5 µM), we hardly see any effect nor in cell viability or in the colony formation assays (**Figure 2** and **Supplementary Figures S1A**–**F**). In addition, our results are consistent with several studies that have shown that selective attenuation of BER by knockdown or inhibition of their components sensitizes cells to PARP inhibition (41, 42). In particular, it has been described that the knockdown of OGG1 conferred sensitivity to PARP1 inhibition (20, 39).

In conclusion, our results encourage us to hypothesize that OGG1 inhibition may represent a potential way to maximize the clinical effectiveness of PARPi, for instance, overcoming the resistance to PARP inhibition, or the unacceptable toxicity frequently reported when PARPi are combined with conventional chemotherapies (12, 43) or by expanding the spectrum of tumors potentially sensitive to PARPi. Complementary, it would also be interesting to evaluate the potential synthetic lethality between *OGG1* and *BRCA2*, given that BRCA2-deficient cells are also highly sensitive to PARP inhibition (3, 44). The preliminary results shown here might represent the proof-of-concept for new alternative or complementary therapies for the treatment of hereditary breast and ovarian cancer. However, future preclinical studies will be needed before bringing the OGG1 inhibitors to the clinic.

## Data Availability Statement

The original contributions presented in the study are included in the article/**Supplementary Material**. Further inquiries can be directed to the corresponding author.

## Author Contributions

Study conception and design: AO, JMB, JB, CB-B. Acquisition of data: JMB, EM-P, RM, RT-R, SR-P, TH. Analysis and interpretation of data: JMB, EM-P, CB-A, AO, JB. Drafting of the manuscript: JMB, AO. All authors read and approved the final manuscript

## Funding

JMB is supported by grant FPU15/01978 from the Spanish Ministry of Education, Culture and Sport. RT-R is supported by a fellowship from the AECC scientific foundation. SR-P is supported by grants from the Spanish National Research and Development Plan, Instituto de Salud Carlos III, and FEDER (PI17/02303, PI20/01837 and DTS19/00111); AEI/MICIU EXPLORA Project BIO2017-91272-EXP and AECC_Lab_2020 Project (Asociación Española Contra el Cáncer). JB’s laboratory is partially funded by FIS PI16/00440 supported by FEDER funds, H2020 BRIDGES project and the Spanish Network on Rare Diseases (CIBERER). AO is partially funded by Instituto de Salud Carlos III, project reference PI19/00640, cofunded by the European Regional Development Fund (ERDF), “A way to make Europe” and the Spanish Network on Rare Diseases (CIBERER).

## Conflict of Interest

TH is listed as inventor on a provisional U.S. patent application no. 62/636983, covering OGG1 inhibitors. The patent is fully owned by a non-profit public foundation, the Helleday Foundation, and TH is member of the foundation board developing OGG1 inhibitors toward the clinic.

The remaining authors declare that the research was conducted in the absence of any commercial or financial relationships that could be construed as a potential conflict of interest.

## Publisher’s Note

All claims expressed in this article are solely those of the authors and do not necessarily represent those of their affiliated organizations, or those of the publisher, the editors and the reviewers. Any product that may be evaluated in this article, or claim that may be made by its manufacturer, is not guaranteed or endorsed by the publisher.
