# Supplementary material for: OGG1 Inhibition Triggers Synthetic Lethality and Enhances The Effect of PARP Inhibitor Olaparib in BRCA1-Deficient TNBC Cells
Source: Front Oncol. 2022 May 10;12:888810. doi: 10.3389/fonc.2022.888810 (PMC9127384; doi:10.3389/fonc.2022.888810)
Supplement: Supplementary file 1 [file DataSheet_1.docx]

**Supplementary data**

**Title:** OGG1 inhibition triggers synthetic lethality and enhances the effect of PARP inhibitor olaparib in *BRCA1*-deficient TNBC cells.

Juan Miguel Baquero^1^, Erik Marchena-Perea^2^, Rocío Mirabet^1^, Raúl Torres-Ruiz^3,4^, Carmen Blanco-Aparicio^5^, Sandra Rodríguez-Perales^3^, Thomas Helleday^6,7^, Carlos Benítez-Buelga^6^, Javier Benítez^1,8,^ and Ana Osorio^1,2,8*^.

^1^Human Genetics Group, Human Cancer Genetics Programme, Spanish National Cancer Research Centre (CNIO), Madrid, 28029, Spain

^2^Familial Cancer Clinical Unit, Human Cancer Genetics Programme, Spanish National Cancer Research Centre (CNIO), Madrid, 28029, Spain

^3^Molecular Cytogenetics Group, Human Cancer Genetics Programme, Spanish National Cancer Research Centre (CNIO), Madrid, 28029, Spain

^4^Division of Hematopoietic Innovative Therapies, Centro de Investigaciones Energéticas, Medioambientales y Tecnológicas (CIEMAT), Madrid, Spain.

^5^Experimental Therapeutics Program. Spanish National Cancer Research Centre (CNIO), Madrid, 28029, Spain

^6^Science for Life Laboratory, Department of Oncology-Pathology, Karolinska Institutet, 171 76 Stockholm, Sweden.

^7^Sheffield Cancer Centre, Department of Oncology and Metabolism, University of Sheffield, Sheffield S10 2RX, United Kingdom

^8^Spanish Network on Rare Diseases (CIBERER), Madrid, 28029, Spain

**SUPPLEMENTARY TABLES**

| **Supplementary Table S1 - List of primers used** | | |
| --- | --- | --- |
| Primer pair | 5'-3' Forward primer | 5'-3' Reverse primer |
| BRCA1-exon11 | AGTTGGTTGATTTCCACCTC | CCAGTGATCCTCATGAGGCT |
| GAPDH-cDNA | CCTGCACCACCAACTGCTTA | CCATCACGCCACAGTTTCC |
| BRCA1-cDNA (exons 12-13) | GAAGCAGCATCTGGGTGTGA | ATTTCGCAGGTCCTCAAGGG |

**Suplemetary Table S2. Analysis of the combined treatment of olaparib+TH5478 and niraparib+SUO268 in MDA-MB-231 and Hs578T parental cells and BRCA1-KOs.**

MDA-MB-231 (cell viability assay)

| Drug combination | BRCA1ko1 | BRCA1ko2 | BRCA1 proficient |
| --- | --- | --- | --- |
| 0.5µM Olaparib + 3.75µM TH5487 | CI=1.00 | CI=1.02 | CI=1.56 |
| 1µM Olaparib + 3.75µM TH5487 | CI=0.98 | CI=1.02 | CI=1.46 |

Hs 578T (cell viability assay)

| Drug combination | BRCA1ko1 | BRCA1ko2 | BRCA1 proficient |
| --- | --- | --- | --- |
| 0.5µM Olaparib + 3.75µM TH5487 | CI=0.94 | CI=0.92 | CI=1.37 |
| 1µM Olaparib + 3.75µM TH5487 | CI=1.02 | CI=0.91 | CI=1.29 |

MDA-MB-231 (clonogenic assay)

| Drug combination | BRCA1ko1 | BRCA1ko2 | BRCA1 proficient |
| --- | --- | --- | --- |
| 30nM Olaparib + 3µM TH5487 | CI=0.78 | CI=0.94 | CI=1.11 |
| 50nM Olaparib + 5µM TH5487 | CI=0.78 | CI=0.98 | CI=1.13 |

Hs578T (clonogenic assay)

| Drug combination | BRCA1ko1 | BRCA1ko2 | BRCA1 proficient |
| --- | --- | --- | --- |
| 30nM Olaparib + 3µM TH5487 | CI=0.71 | CI=0.93 | CI=1.43 |
| 50nM Olaparib + 5µM TH5487 | CI=0.85 | CI=0.94 | CI* |

Hs578T (viability assay)

| Drug combination | BRCA1ko1 | BRCA1ko2 | BRCA1 proficient |
| --- | --- | --- | --- |
| 13µM Niraparib  + 2.5µM SUO268 | Not performed | CI=0.78 | CI=0.65 |
| 13µM Niraparib  + 5µM SUO268 | Not performed | CI=0.51 | CI=0.42 |

*CI could not be calculated for this dose combination due to technical problems with the experiment.

**SUPPLEMENTARY FIGURES**


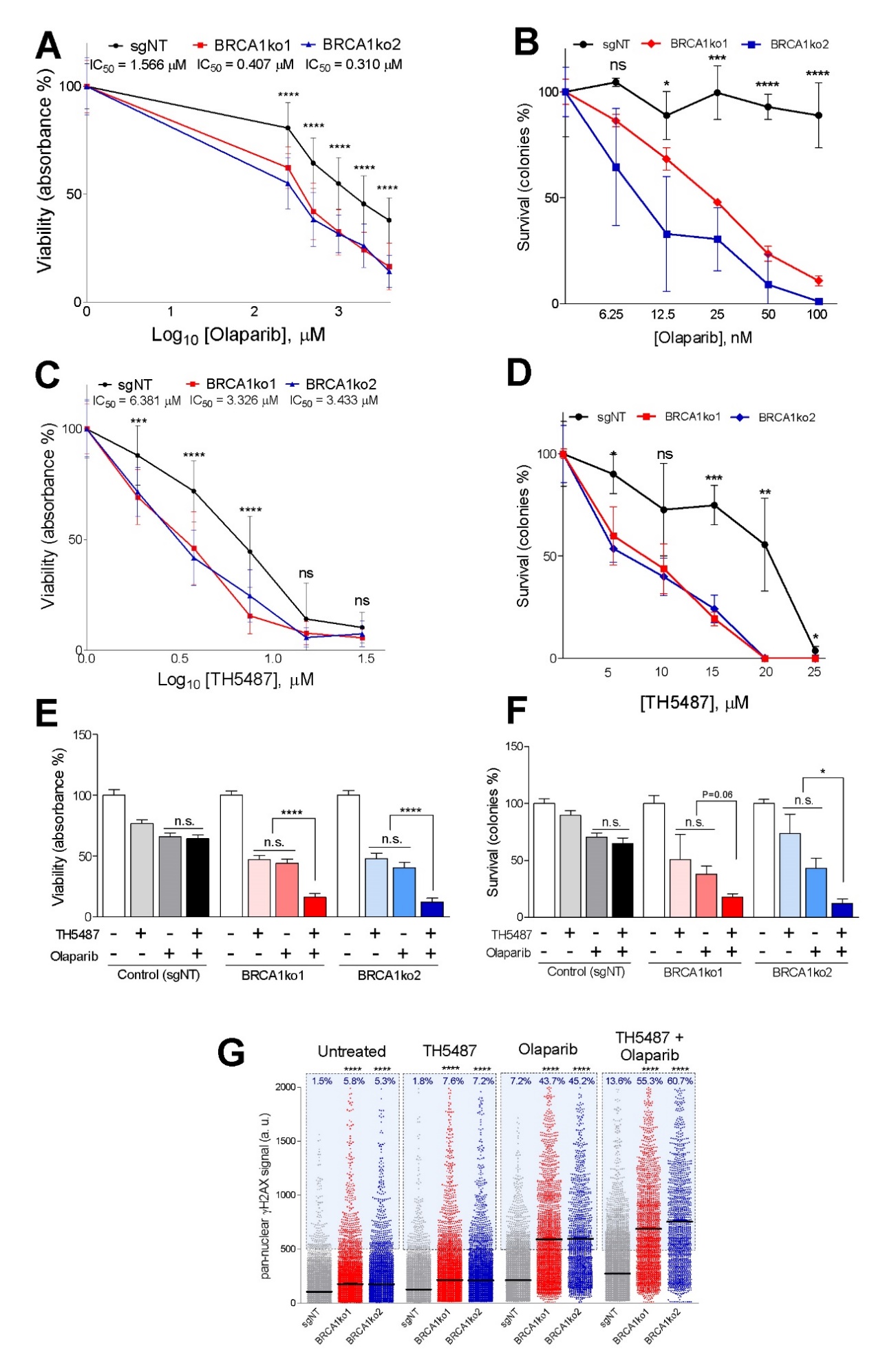

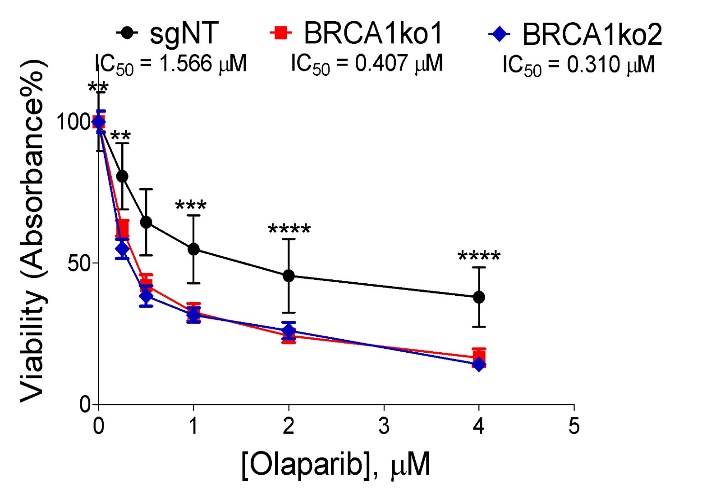


**A**


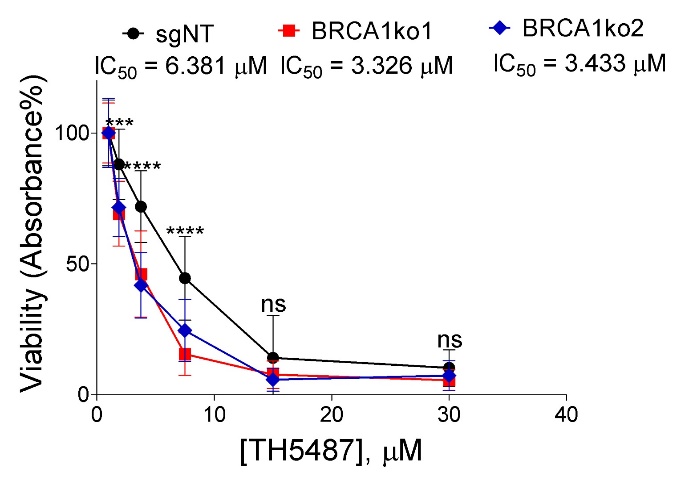


**C**

**Supplementary Figure 1: Results validation in Hs 578T TNBC cell line. A)** MTT assay displaying logarithm-transformed values and the viability curves of BRCA1-proficient (sgNT) and deficient (BRCA1ko1 and BRCA1ko2) Hs 578T cells after treatment with olaparib for 72 hours. **B)** Clonogenic survival of BRCA1-proficient (sgNT) and deficient (BRCA1ko1 and BRCA1ko2) Hs 578T cells exposed to olaparib. **C)** MTT assay displaying drug dose values and the viability curves of BRCA1-proficient (sgNT) and deficient (BRCA1ko1 and BRCA1ko2) Hs 578T cells after treatment with TH5487 for 72 hours. **D)** Clonogenic survival of BRCA1-proficient (sgNT) and deficient (BRCA1ko1 and BRCA1ko2) Hs 578T cells exposed to TH5487. **E)** Cell viability assessment using MTT displaying proliferation changes of BRCA1-proficient (sgNT) and deficient (BRCA1ko1 and BRCA1ko2) Hs 578T cells after single-drug (TH5487 3.75 µM or olaparib 0.5 µM) or combined treatments for 72 hours. **F)** Clonogenic survival of BRCA1-proficient (sgNT) and deficient (BRCA1ko1 and BRCA1ko2) Hs 578T cells exposed to TH5487 (3 µM), olaparib (30 nM), or a combination of both inhibitors. **G)** Pan-nuclear γH2AX signal intensity of BRCA1-proficient (sgNT) and deficient (BRCA1ko1 and BRCA1ko2) Hs 578T cells exposed to TH5487 (3.75 µM), olaparib (0.5 µM), or a combination of both. In (A) and (C) for each concentration were included six replicates in at least two independent plates. IC_50_ calculated based on the resulting dose-response curves are shown. In (B) and (D) cells were incubated for 14 days in the presence of DMSO (control) or the indicated concentrations of olaparib or TH5487 in three independent experiments. In (E) and (F) values are normalized to untreated cells (DMSO) and bars represent the mean and the SEM of at least three independent experiments. In (G) each dot represents the signal from one cell, horizontal lines indicate mean values, and the blue area delineates cells above an arbitrarily chosen threshold. Each condition includes at least 2000 cells from 3 independent experiments. In (A), (B), (C), and (D) statistical significance at each olaparib or TH5487 concentration was determined by a one-way ANOVA test. The values are normalized to untreated cells and error bars represent one standard deviation around the mean. Unpaired t‐tests were used in (E) and (F). In (G) statistical significance was determined by Mann-Whitney U tests.

**
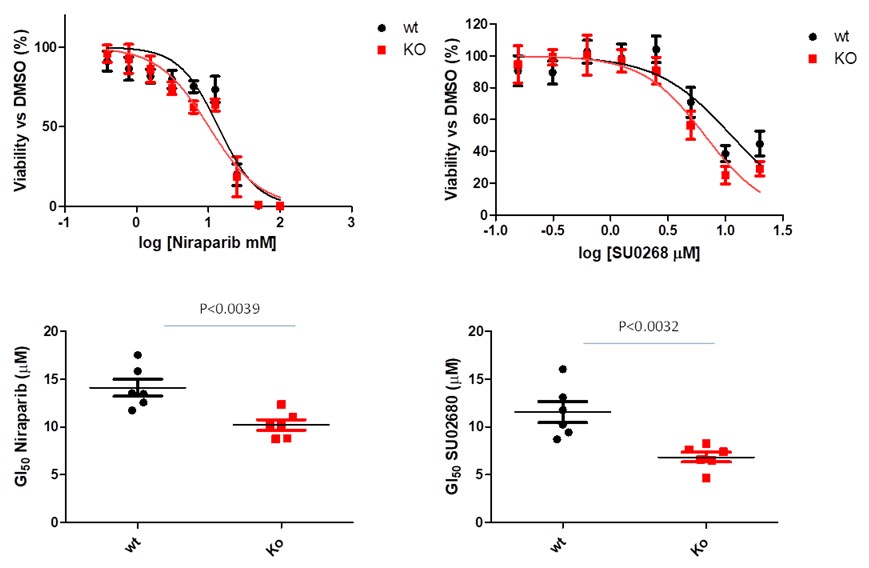
**

**Supplementary Figure 2**: MTT assays displaying logarithm-transformed values and the viability curves of BRCA1-proficient (WT) and deficient (KO) Hs 578T cells after treatment with SUO268 for 72 hours. The lower graph represents the differences between IC50 values between WT and KO cell lines for SUO268.
